# Comparative proteomic analysis identified proteins and the phenylpropanoid biosynthesis pathway involved in the response to ABA treatment in cotton fiber development

**DOI:** 10.1038/s41598-023-28084-3

**Published:** 2023-01-27

**Authors:** Yong Yang, Wenjie Lai, Lu Long, Wei Gao, Fuchun Xu, Ping Li, Shihan Zhou, Yuanhao Ding, Haiyan Hu

**Affiliations:** 1 Hainan Key Laboratory for Sustainable Utilization of Tropical Bioresources, College of Tropical Crops, Hainan University, Haikou, 570228 China; 2 Hainan Yazhou Bay Seed Laboratory, Sanya Nanfan Research Institute of Hainan University, Sanya, China; 3 State Key Laboratory of Cotton Biology, School of Life Sciences, Henan University, Kaifeng, China

**Keywords:** Plant hormones, Plant signalling, Secondary metabolism

## Abstract

Abscisic acid (ABA) is a plant hormone that plays an important role in cotton fiber development. In this study, the physiological changes and proteomic profiles of cotton (*Gossypium hirsutum*) ovules were analyzed after 20 days of ABA or ABA inhibitor (ABAI) treatment. The results showed that compared to the control (CK), the fiber length was significantly decreased under ABA treatment and increased under ABAI treatment. Using a tandem mass tags-based quantitative technique, the proteomes of cotton ovules were comprehensively analyzed. A total of 7321 proteins were identified, of which 365 and 69 differentially accumulated proteins (DAPs) were identified in ABA versus CK and ABAI versus CK, respectively. Specifically, 345 and 20 DAPs were up- and down-regulated in the ABA group, and 65 and 4 DAPs were up- and down-regulated in the ABAI group, respectively. The DAPs in the ABA group were mainly enriched in the biosynthesis of secondary metabolites, phenylpropanoid biosynthesis and flavonoid secondary metabolism, whereas the DAPs in the ABAI group were mainly enriched in the indole alkaloid biosynthesis and phenylpropanoid biosynthesis pathways. Moreover, 9 proteins involved in phenylpropanoid biosynthesis were upregulated after ABA treatment, suggesting that this pathway might play important roles in the response to ABA, and 3 auxin-related proteins were upregulated, indicating that auxin might participate in the regulation of fiber development under ABAI treatment.

## Introduction

Upland cotton (*Gossypium hirsutum*), as the most important fiber crop, is a main source of raw materials for textile production worldwide. Cotton fiber is a single epidermal cell derived from the cotton seed coat. The differentiation and development of cotton fiber cells undergo five overlapping stages: initiation, elongation, transition, secondary cell wall (SCW) deposition and maturation^1^. It has been reported that the plant hormone auxin was regulated by overexpression of *GhiaaM* to promote fiber cell initiation and development, and gibberellin triggered the release of the GhHOX3 protein to affect fiber cell elongation^2,3^. Brassinosteroids was regulated by the expression of *GhDET2*, which was involved in fiber initiation and elongation, and the jasmonic acid signaling pathway was negatively regulated by *GhJAZ2* to mediate fiber initiation and elongation^4,5^. Abscisic acid (ABA) was also a key plant hormone that inhibited fiber initiation and elongation^6^. However, the molecular mechanism of ABA regulation and the regulatory network of the ABA signaling pathway in cotton fiber development are still unclear.

ABA is a major phytohormone that regulates the expression of many genes, leading to important physiological and biochemical changes for survival under stress^7^. Previous studies showed that the accumulation of ABA reached its first peak during fiber elongation and SCW deposition, and a second peak occurred at 37 DPA (days postanthesis)^8–10^. During fiber growth, ABA affected the leakage and uptake of potassium from the ovules^11^. Moreover, the application of exogenous ABA could rescue the browning of ovules caused by calcium deficiency^12^. Therefore, ABA plays an important role in the development of cotton fiber.

During fiber development, there was a transition stage from elongation to SCW deposition at 16 DPA^10,13^. The onset of SCW deposition gradually terminated cotton fiber elongation which was regulated by primary cell wall extension^14–16^. The phenylpropanoid pathway participated in lignin deposition in the plant cell wall, resulting in cell growth cessation and reduced plasticity^17^. Phenylpropanoid biosynthesis is one of the most important secondary metabolic pathways, including coumarins, flavonoids, stilbenes, hydroxycinnamates, lignans and the macromolecule lignin^18^. The pathway mainly contained phenylalanine ammonia-lyase (PAL), cinnamate 4-hydroxylase (C4H), 4-coumarate: CoA ligase (4CL), hydroxycinnamoyl-transferase (HCT), P-coumaroyl shikimate 3′ hydroxylase, caffeoyl CoA 3-O-methyltransferase (CCoAOMT), cinnamoyl-CoA reductase (CCR), ferulate 5-hydroxylase (F5H), caffeic acid/5-hydroxyferulic acid O-methyltransferase (COMT) and cinnamyl alcohol dehydrogenase (CAD)^19–24^. Transcriptome analyses showed that phenylpropanoid pathway genes including 4CL, C4H, COMT, CCR and CAD, were preferentially expressed during cotton fiber development^25^. Additionally, the content of IAA was also found to increase during the transition stage^26^. Auxin accumulation was regulated by an auxin transporter encoded by *GhPIN3a* in fiber cells^27^. In contrast to promoting fiber elongation, increased auxin at this stage promoted the expression of *GhRAC13* and induced secondary wall formation through reactive oxygen species^28,29^. This may result from the premature deposition of SCW that limits cell elongation in the fiber^30^.

To further investigate the molecular regulatory mechanisms of cotton ovules in response to ABA, a comparative proteomics analysis of ovules after culture with ABA and diphenyliodonium chloride (ABA inhibitor, ABAI) for 20 days was performed using a tandem mass tag (TMT) quantitative proteomics technique. This research aimed to investigate the proteins involved in the response to ABA and explore the potential molecular mechanisms of cotton fiber development. Our findings may provide a comprehensive proteome reference for further investigation of the roles of differentially accumulated proteins (DAPs) in cotton fiber development.

## Results

### ABA negatively regulates fiber expansion in ovules of *G. hirsutum*

**Figure 1:**
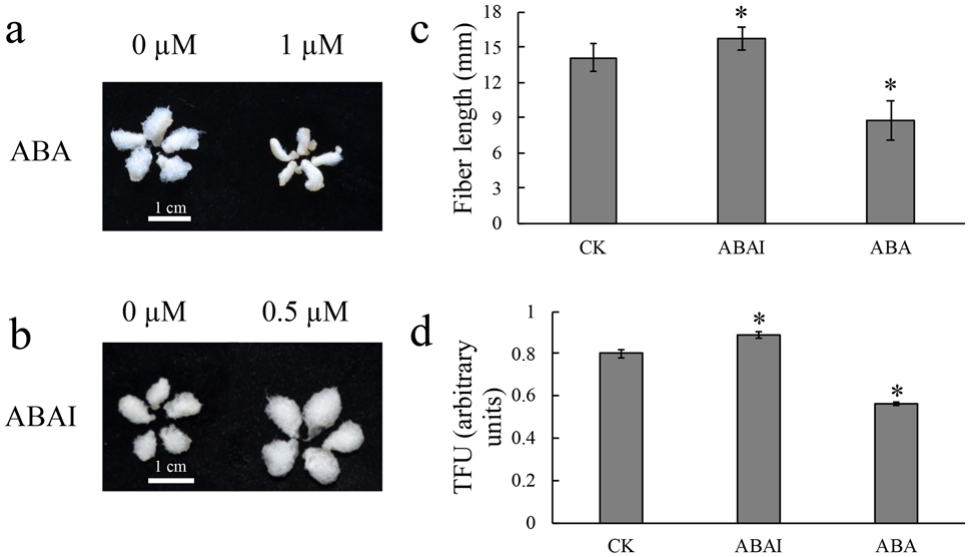
The 0 DPA ovules were cultured in BT medium with ABA and ABAI for 20 days. (**a**, **b**) Phenotypes of cotton ovules under ABA or ABAI treatment. Bar = 1 cm. (**c**) Fiber length on the cultured ovules in different treatments. (**d**) Total fiber units on cultured ovules under different treatments. *Indicates a significant difference with *p* < 0.05 using a *t test*.

To evaluate the effect of ABA and ABAI on fiber elongation, 0 DPA ovules were used for in vitro culture, and the phenotype of cotton ovules was recorded after 20 days. The fiber length was inhibited when ovules were cultured in BT medium supplemented with 1 µM ABA and promoted when ovules were cultured in BT medium supplemented with 0.5 µM ABAI (Fig. 1A). Fiber length was only 62.1% of the CK after ABA treatment and increased by 11.6% after ABAI treatment (Fig. 1B). Moreover, the TFU (total fiber units) in ABA group was lower than that in the CK group, while higher in the ABAI group (Fig. 1C). Therefore, fiber development was inhibited by exogenous ABA and was promoted by ABAI.

### TMT-based quantitative proteomics analysis and protein identification

**Figure 2:**
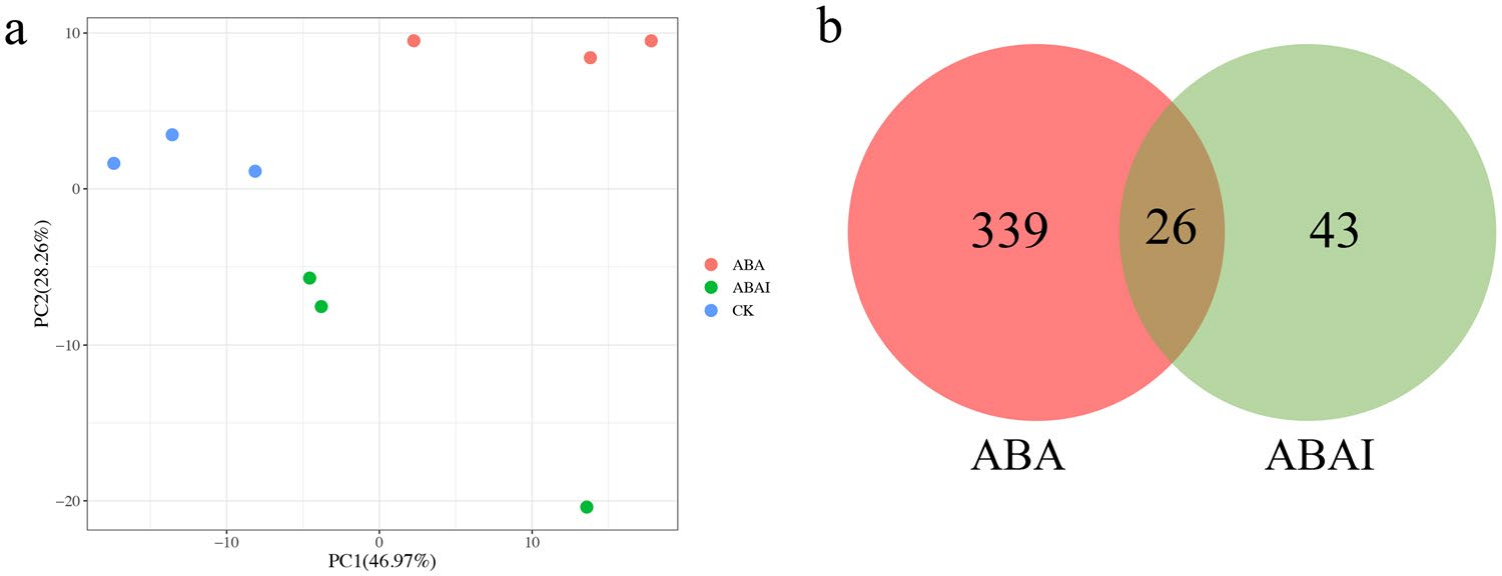
Overview of quantitative proteomic data. (**a**) PCA of proteins in the CK, ABA and ABAI groups. (**b**) Venn diagram of DAPs in cotton ovules treated with ABA and ABAI.

To explore the difference in the mechanism of cotton ovules after ABA and ABAI treatment, TMT-based quantitative proteomics was performed among the three groups, CK, ABA and ABAI. These ovule samples were carefully ground for protein extraction, digestion, TMT 2D-LC‒MS/MS analysis and quantification. A total of 69,176 peptides were identified through spectrum analysis, and 24,287 unique peptides were identified. A total of 7321 reliable proteins were identified in all the samples with unique peptides ≥ 1 (Fig. S1). Principal component analysis showed sufficient reproducibility of the experiment, with the three groups showing clear separation (Fig. 2A). Compared with the CK group, 365 DAPs were identified after ABA treatment, including 345 highly accumulated proteins and 20 lowly accumulated proteins, of which 339 proteins were unique ABA-response proteins (Fig. 2B). In ABAI versus CK, 69 proteins were differentially accumulated, with 65 upregulated and 4 downregulated proteins, of which 43 showed specific accumulation patterns. Furthermore, 26 DAPs were commonly shared in both the ABA and ABAI groups (Figs. 2B, S2, S3).

### DAPs were mainly enriched in phenylpropanoid and auxin biosynthesis

All the DAPs (365 and 69) were annotated by GO and KEGG pathway analysis to further understand their functions, and the details are summarized in Tables S2 and S3. To characterize the functions of the DAPs, the data were subjected to GO enrichment. For ABA treatment, the biological progress (BP) analysis indicated that DAPs were mainly involved in single-organism metabolic process, single-organism biosynthetic process and phenylpropanoid biosynthetic process; cytoplasm, cytoplasmic part and cell periphery were the top three GO terms in cellular component (CC); while catalytic activity and oxidoreductase activity were found for molecular function (MF) (Fig. S4). For ABAI treatment, BP analysis revealed that DAPs were mainly enriched in response to oxygen-containing compound, secondary metabolic process and regulation of response to stimulus term; cell, cell part and cytoplasm were the top three terms in CC; while auxin transmembrane transporter activity and auxin efflux transmembrane transporter activity were enriched in MF (Fig. S4).

**Figure 3:**
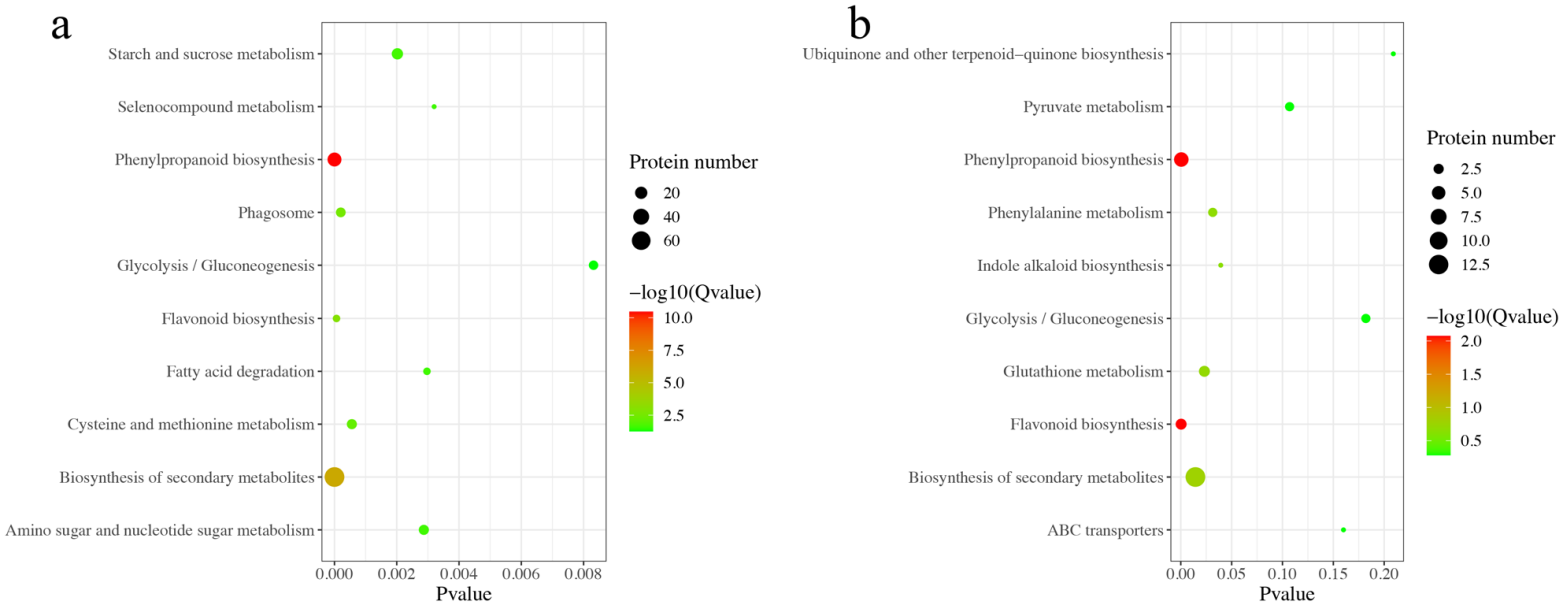
KEGG pathway enrichment of DAPs under ABA (**a**) and ABAI (**b**) treatment.

KEGG pathway analysis indicated that the DAPs were enriched in 76 KEGG pathways (Table S4), and the top 10 pathways were investigated (Fig. 3A), revealing that several metabolic processes, including the biosynthesis of secondary metabolites, starch and sucrose metabolism, and phenylpropanoid biosynthesis, were greatly influenced after ABA treatment. After ABAI treatment, the DAPs were enriched in 34 pathways (Table S5); the top 10 pathways were shown in Fig. 3B. The DAPs were most associated with the biosynthesis of secondary metabolites, flavonoid biosynthesis, phenylpropanoid biosynthesis and indole alkaloid biosynthesis. In summary, a large number of proteins involved in phenylpropanoid biosynthesis were upregulated under ABA treatment, while DAPs involved in phenylpropanoid biosynthesis and indole alkaloid biosynthesis were upregulated under ABAI treatment. The results provided important information to elucidate the development of cotton ovules in response to ABA.

### Interaction network analysis of DAPs

**Figure 4:**
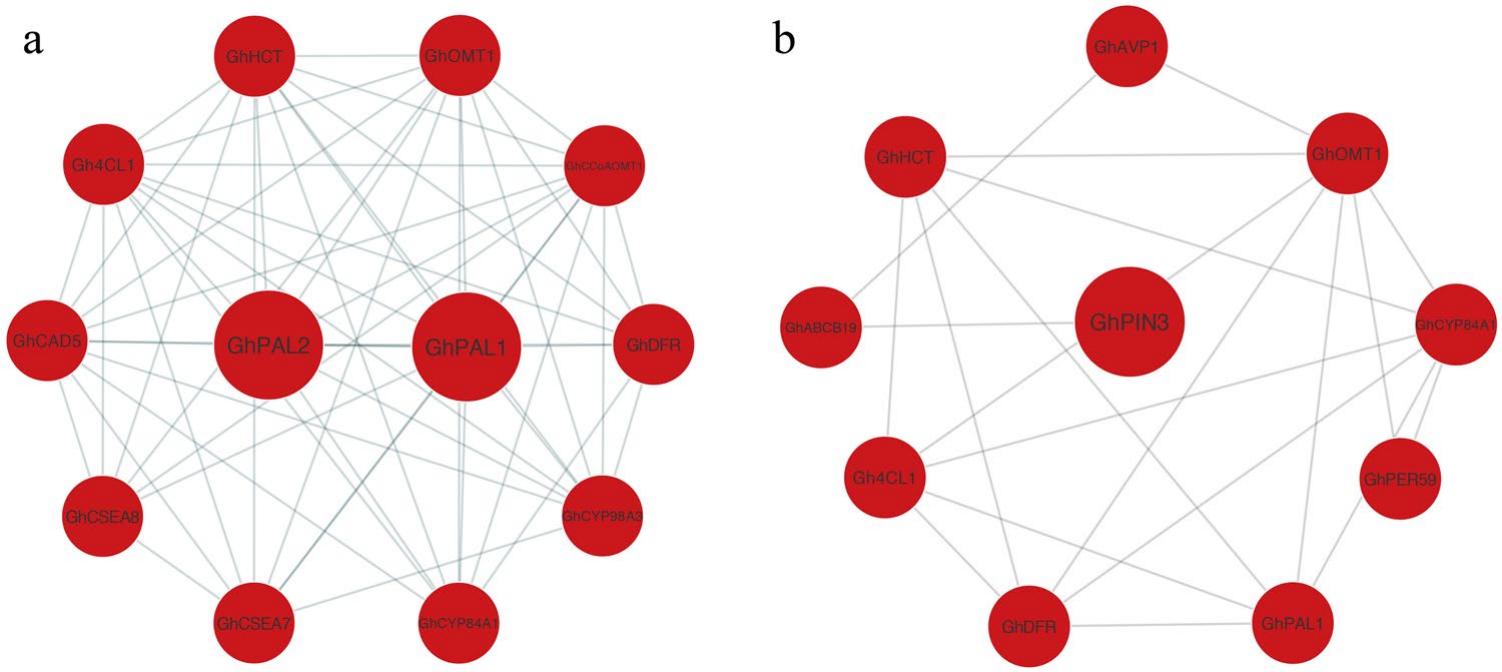
Protein‐protein interaction (PPI) network analysis of DAPs. (**a**) The top PPI network using MCODE analysis under ABA treatment. (**b**) The top PPI network under ABAI treatment.

To better understand the protein regulatory network, we used the DAPs for PPI network analysis via the STRING database. The ABA protein interaction module was screened by MCODE, and the DAPs constituted a complex and strongly interactive network. In the network, there were 12 nodes and 57 edges, of which PAL1 and PAL2 were the initiating enzymes that regulated the phenylpropanoid synthesis pathway (Fig. 4A), which catalyzed the first step in the phenylpropanoid metabolism pathway^18^. In the ABAI PPI network, there were 10 nodes and 10 edges (Fig. 4B). Among the 10 proteins, the protein PIN3 was an auxin efflux carrier family protein. As an important signaling molecule, auxin is deeply involved in the growth and development of cotton fiber^31,32^.

### Validation by mRNA expression levels

**Figure 5:**
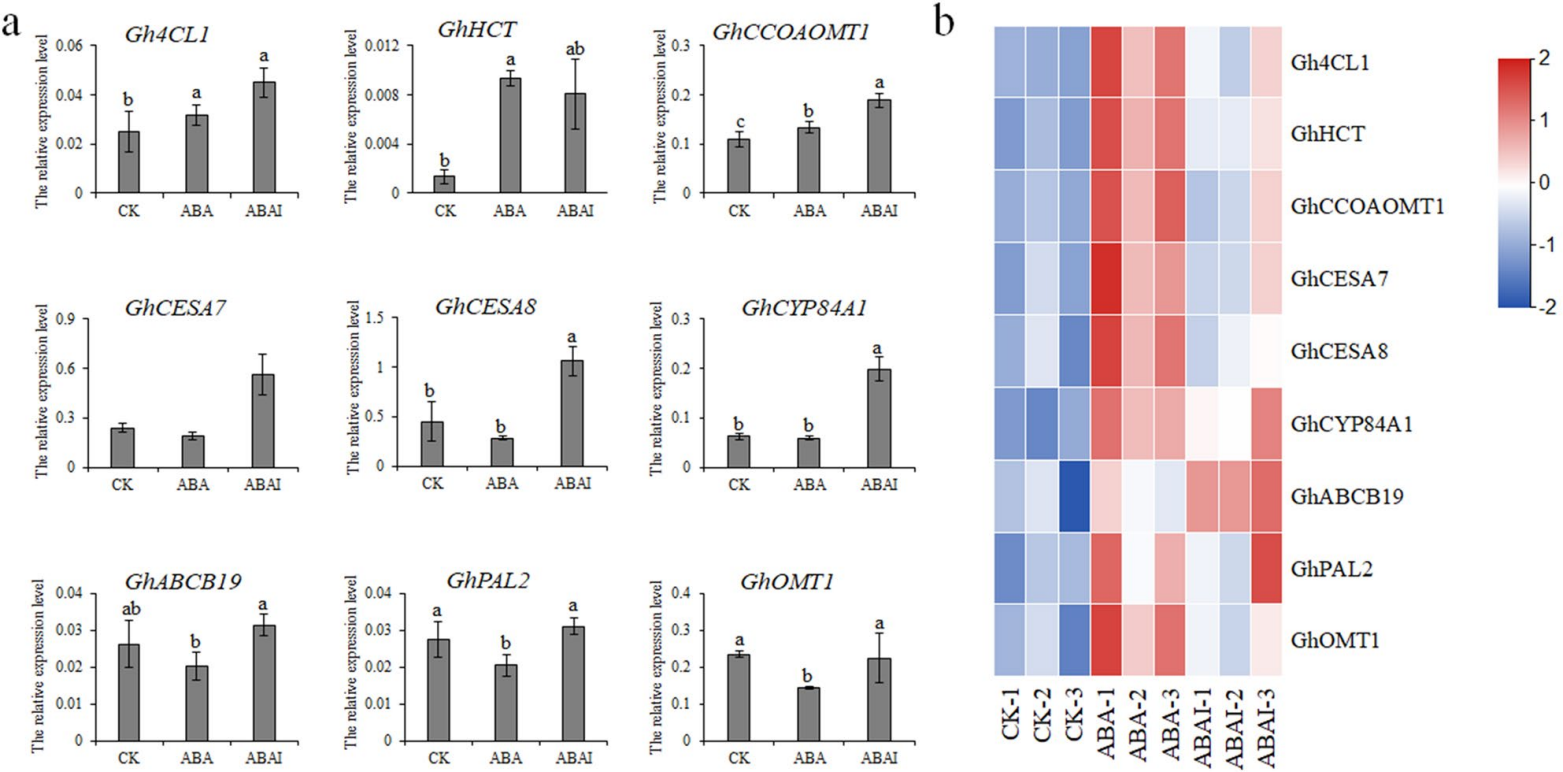
Comparison of the different expression levels of 9 DAPs using qRT-PCR results and proteomic data in the CK, ABA and ABAI groups. (**a**) Nine DAPs were selected to examine the mRNA expression level by qRT‐PCR analysis. (**b**) The heatmap was generated using the relative content of 9 DAPs by TMT analysis. Different letters indicate significant differences between groups (*p* < 0.05).

To examine whether the gene expression levels were correlated with the protein levels, qRT-PCR was used to analyze the expression levels of 9 genes under ABA or ABAI treatment. Eight proteins were associated with phenylpropanoid metabolism, including Gh_D10G0473 (Gh4CL1), Gh_A05G1005(GhHCT), Gh_A13G0363 (GhCCoAOMT1), Gh_A05G3965 (GhCESA7), Gh_D10G0333 (GhCESA8), Gh_A11G1648(GhCYP84A1), Gh_D10G2528 (GhPAL2) and Gh_D08G1135 (GhOMT1). One protein, Gh_D09G0181(GhABCB19), is homologous to AtABCB19, which was an auxin transporter in *Arabidopsis*^33^. The qRT-PCR results showed that 6 selected genes (*Gh4CL1*, *GhHCT*, *GhCCoAOMT1*, *GhCESA7*, *GhCESA8* and *GhCYP84A1*) were upregulated in accordance with the abundance changes of proteins, distributed in the phenylpropanoid biosynthesis pathway (Fig. 5A,B). As reported previously, alternative splicing, mRNA stability and translation may result in the differences between the expression of mRNA and protein^34^. Three genes (*GhPAL2*, *GhOMT1* and *GhABCB19*) showed partially consistent results with the expression levels of the proteins, indicating a complex regulatory phase from the transcriptome to the proteome.

## Discussion

Cotton is cultivated in many countries and the fibers are important natural materials in the textile industry. Phytohormones have been shown to be involved in the development of fiber cells in cotton^35^. However, a comprehensive understanding of how the ABA signaling pathway participates in fiber cell development is still lacking. Our results showed that exogenous ABA inhibited fiber cell elongation, and the inhibition was correlated with an increase in ABA concentration, which is consistent with a previous study^6^. Moreover, the inhibition of ABA can promote the fiber cell development (Fig. 1). The results suggested that ABA negatively regulates the fiber cell development.

To further elucidate the molecular network in response to ABA, the proteomic data from ABA and ABAI treatments were analyzed. We found that 345 of 365 DAPs in the ABA groups were upregulated and 65 of 69 DAPs in the ABAI groups were upregulated, while only 26 of the DAPs were shared, suggesting that protein responses might differ in the presence and absence of ABA. By KEGG categorization, a proportion of proteins involved in the biosynthesis of secondary metabolites such as phenylpropanoids and flavonoids were activated under ABA or ABAI treatment. Because phenylpropanoids are key indicators of plant responses to hormones, we further analyzed the DAPs involved in the phenylpropanoid biosynthesis pathway^36,37^.

The phenylpropanoid pathway plays important roles in the fiber developmental transition from elongation to SCW deposition^38^. In our study, GhPAL1, GhPAL2, Gh4CL1, GhCCoAOMT1, GhOMT1, GhCYP98A3 and GhCAD5 were enriched in the phenylpropanoid pathway and were upregulated in response to ABA treatment. PAL-box genes associated with lignin biosynthesis were found to be activated by *GhWLIM1a* to form SCW^39^. *GhCAD1* and *GhCAD6* were found to be involved in SCW formation^40^. And *GhCesA7*, *GhCesA8*, *GhPAL1*, *GhF5H* and *Gh4CL1* were upregulated by overexpression of *GhMYBL1*, resulting in SCW thickening^41^. The results suggested that the phenylpropanoid pathway was activated by ABA. Moreover, GhCesA7 and GhCesA8 were also upregulated in response to ABA. Cellulose synthases (*GhCesA4*, *GhCesA7* and *GhCesA8*) were found to play important roles in SCW cellulose deposition in cotton fiber^42,43^. Therefore, these results suggested that an early onset of the SCW thickening process might be activated by the phenylpropanoid biosynthesis pathway under ABA treatment.

Auxin plays an important role in cotton fiber yield and fitness^2^. In the cotton ovule epidermis, polar auxin transport participated in establishing a gradient via *GhPIN*^27^. The inhibition of *GhPINs* significantly suppressed the initiation and elongation of cotton fibers. Moreover, *GhPIN1a_Dt*, *GhPIN6_ At*, and *GhPIN8_At* were preferentially expressed in the fiber initiation and elongation stages, and overexpression of these genes increased the density and length of the leaf trichomes in *Arabidopsis*^44^. *AtABCB19*, an auxin efflux transporter, was found to be involved in lateral root development with *AtPIN2*, *AtPIN3* and *AtPIN7*^45^. Overexpression of *AtAVP1* simulates auxin transport and *AVP1* expression in cotton increases fiber yield under stress^46^. The hormonal balance of auxin and ABA was reported to be correlated with the abscission of cotton fruits^47^. Recently, auxin was found to promote the transition from the elongation stage to the second wall deposition stage during cotton fiber development^29^. In our data, 3 auxin-related proteins were upregulated under ABAI treatment, including Gh_A01G1254 (GhPIN3), GhABCB19 and Gh_A05G0066 (GhAVP1), indicating that the synthesis of indole acetic acid might be promoted. Thus, we speculated that the indole alkaloid biosynthesis pathway might be activated with the increased accumulation of these proteins in response to ABAI, and auxin may be involved in the regulation of signal transmission in fiber cell development after the inhibition of ABA.

## Conclusion

In this study, the application of exogenous ABA resulted in the inhibition of cotton fiber development, and the application of ABAI promoted the fiber development. Moreover, we provided a comprehensive understanding of the molecular mechanism of cotton fiber development in response to ABA by using ABA and ABAI treatment. A total of 7321 proteins were identified, including 365 and 69 DAPs in ABA versus CK and ABAI versus CK, respectively. Proteomic analysis showed significant proteome changes in phenylpropanoid biosynthesis and flavonoid secondary metabolism in the ABA group, suggesting that ABA might be involved in SCW biosynthesis. Moreover, the enrichment of indole alkaloid biosynthesis might promote fiber elongation in the ABAI group. Taken together, our work presents the first proteomic study of cotton fiber development in response to ABA, which provides new clues to reveal the molecular mechanism by which hormones regulate fiber development.

## Methods

### Plant materials and culture in vitro

Cotton plants (*Gossypium hirsutum* TM-1) were grown in the greenhouse at Hainan University in Haikou, Hainan, China. The 0 DPA ovules were collected from the flower buds at 9:00–10:00 AM. Bolls were sterilized with 8% sodium hypochlorite (NaOCl) for 10 min and washed with sterile water for 3 times. Then, the ovules were carefully picked out and cultured in the dark on the surface of liquid BT medium according to the previous reports^48^. On the basis of our pre-experiment treatment with different concentrations of ABA and ABAI, 1 µM ABA (Sigma, Cat. No. 90769, USA) and 0.5 µM ABAI (Sigma, Cat. No. 43088, USA) was supplemented in the BT medium at 25 °C for 20 days. The treated cotton ovules were immediately frozen in liquid nitrogen and stored at − 80 °C until use. The plants and procedures used in the study were carried out according to the guidelines and legislation of Hainan, China.

### Determination of fiber length and TFU (total fiber units)

The fiber length and TFU were measured according to previously described methods^49,50^. Briefly, the cultured ovules were dispersed in boiling water, and then the dried fibers were stained in 0.02% toluidine blue for 30 s and washed three times in deionized water. The samples were eluted in glacial acetic acid:ethanol:water (10:95:5) for 2 h and the absorbance was measured at 624 nm using a T6 UV–visible spectrophotometer (Beijing Persee, China). There were three replicates with five ovules per replicate per treatment.

### Protein extraction, trypsin digestion and TMT labeling

Three biological replicates from each group were used for the proteomic study. The phenol extraction method was used to prepare the proteins from the fiber samples according to a previous report^51^. The proteins in 0.1 g of sample powder were precipitated by adding 10 mL of acetone containing 10% trichloroacetic acid. The sediment was collected in a new tube and dissolved in lysis solution, and the protein concentration was quantified using the BCA method^52^. Then, the collected protein solution was diluted with triethyl ammonium bicarbonate (TEAB) to a final concentration of 1 g/L. 5 µL of 200 mM Tris(2-carboxyethyl) phosphine was added to the samples, which were then incubated at 55 °C for 1 h, and 5 µL of 375 mM iodoacetamide was added, followed by incubation for 30 min in the dark at room temperature. Approximately 600 µL acetone was added to the samples and then precipitated overnight. The mixture was centrifuged at 8000×*g* for 10 min at 4 °C, and the filtrate was discarded. Approximately 100 µL of 100 mM TEAB was added to resuspend the protein precipitate. Finally, trypsin was added to the protein suspensions to a final quality ratio of 1:40, and samples were incubated at 37 °C overnight. After digestion, the peptide (100 µg) were labeled with the TMT 6 plex Label Reagents (Thermo Scientific, USA) according to the manufacturer’s protocols. Then, the mixtures were incubated for 1 h at room temperature and vacuum dried.

### 2D-LC-MS /MS analysis

Prior to reverse-phase liquid chromatography (RPLC), the labeled peptides were purified by the Agilent 1200 HPLC System (Agilent, USA). The online Nano-RPLC was employed on the Easy-nLC 1000 System (Thermo Scientific, USA). The samples were loaded on a PepMap100 nanoLC trap column (75 μm × 20 mm, 3 μm-C18, NanoViper, Thermo Fisher Dionex) and then washed with Nano-RPLC Buffer (0.1% formic acid and 2% acetonitrile) at a flow rate of 2 μL min^−1^ for 10 min. The peptides were analyzed with an elution gradient of 5–35% acetonitrile (containing 0.1% formic acid) for 70 min on a PepMap100 column (75 μm × 150 mm, 2 μm-C18, NanoViper, Thermo Fisher Dionex).

MS/MS was performed with a Thermo Scientific Q Exactive System combined with a Nanospray device. A data-dependent top-20 analysis method was applied with 70 K resolution for the full MS scans and 17.5 K resolution for high energy collisional dissociation MS/MS scans. Dynamic exclusion was set at 30 s. Full MS scans were conducted in the range of *m*/*z* 300–1800 and 70 K resolution was set at *m*/*z* 200 in the Orbitrap mass analyzer. The 20 most intense peaks were fragmented with a charge state ≥ 2 in a high energy collisional dissociation cell with a normalized collision energy of 27%. The tandem mass spectra were collected with a mass resolution of 35 K at *m*/*z* 200.

### Protein identification and bioinformatics analysis

Protein identification and quantification were analyzed using Proteome Discoverer™ 1.4 software (Thermo Scientific, USA). A database search was performed using SEQUEST with the following parameters: carbamidomethylation of cysteine and TMT isobaric labeling of lysine as static modifications, a precursor mass tolerance of 10 ppm and 0.02 Da fragment tolerance. TMT labeling of the peptide and protein N-termini, deamidation of asparagine and glutamine, methionine oxidation, and formation of pyroglutamate from glutamine on the peptide N-terminus were considered dynamic modifications. Data were searched against a cotton database with a 1% false discovery rate. Furthermore, DAP was defined as at least 1 unique peptide based on the criteria of a fold change ≥ 1.3 or ≤ 0.79 in this study.

Gene Ontology (GO) analysis was performed using the David and QuickGO databases to identify the functional annotation^53,54^. The Kyoto Encyclopedia of Genes and Genomes (KEGG) database was used to investigate biological pathways^55^. The String database was used to predict the protein–protein interaction (PPI) network, and the clustered molecules were visualized by Cytoscape^56,57^.

### RNA isolation and qRT-PCR analysis of DAPs in cotton ovules

Total RNA was extracted from cotton ovules of the CK, ABA and ABAI groups using an RNA-prep Pure Plant Plus Kit (#DP441, Tiangen, Beijing, China). cDNA was synthesized by reverse transcription with FastKing RT SuperMix (TIANGEN, Beijing, China) according to the manufacturer’s instructions. qRT-PCR was performed using a QuanStudio 7 Flex system (Thermo Fisher Scientific, MA, USA) with the following reaction steps: 95 °C for 30 s and 40 cycles of 95 °C for 10 s and 60 °C for 30 s. Each sample was analyzed with three replicates. *GhUBI7* was used as a control for PCR analysis. The primers are shown in Table S1.

### Statistical analysis

Statistical analyses were performed using statistical software SPSS v.26 and Excel 2019 spreadsheet. Statistical significance of the differences in fiber length and TFU were evaluated by using the two-tailed *t test*. One-way ANOVA followed by Waller-Duncan’s test was used for the gene expression analysis. Probability levels of *p* ≤ 0.05 were considered statistically significant.

## Supplementary Information

Supplementary Tables.

Supplementary Figures.

## Data Availability

The data supporting the findings of this study are available within the article and its Supplementary Information.
