# Supplementary material for: Comparative proteomic analysis identified proteins and the phenylpropanoid biosynthesis pathway involved in the response to ABA treatment in cotton fiber development
Source: Sci Rep. 2023 Jan 27;13:1488. doi: 10.1038/s41598-023-28084-3 (PMC9883468; doi:10.1038/s41598-023-28084-3)
Supplement: Supplementary file 2 — Supplementary Figures. [file 41598_2023_28084_MOESM2_ESM.docx]

**
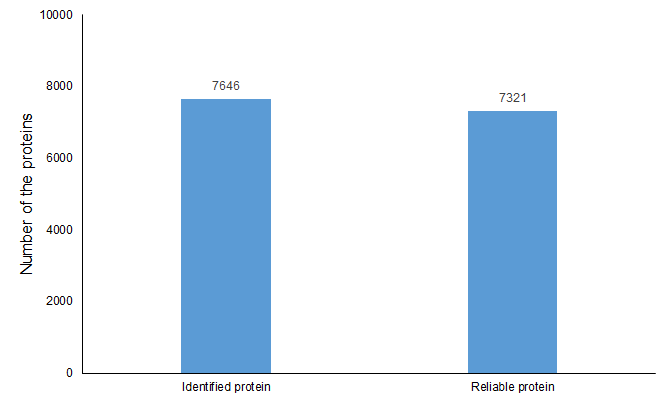
**

**Fig. S1** Number of the unique peptides identified in this study.


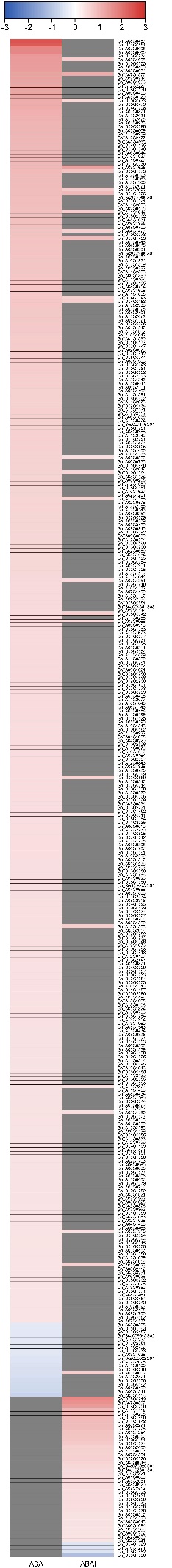


**Fig. S2** Heatmap of the DAPs in ABA and ABAI treatments using TBtools [1].


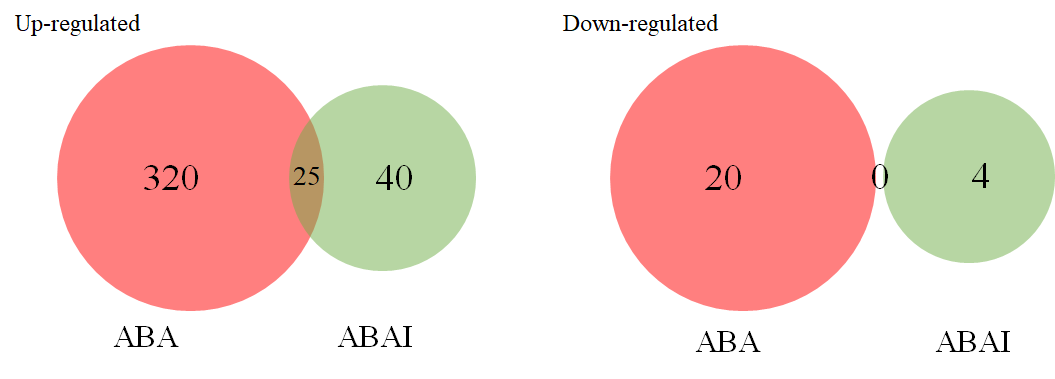


**Fig. S3** Venn diagram of the upregulated and downregulated DAPs.


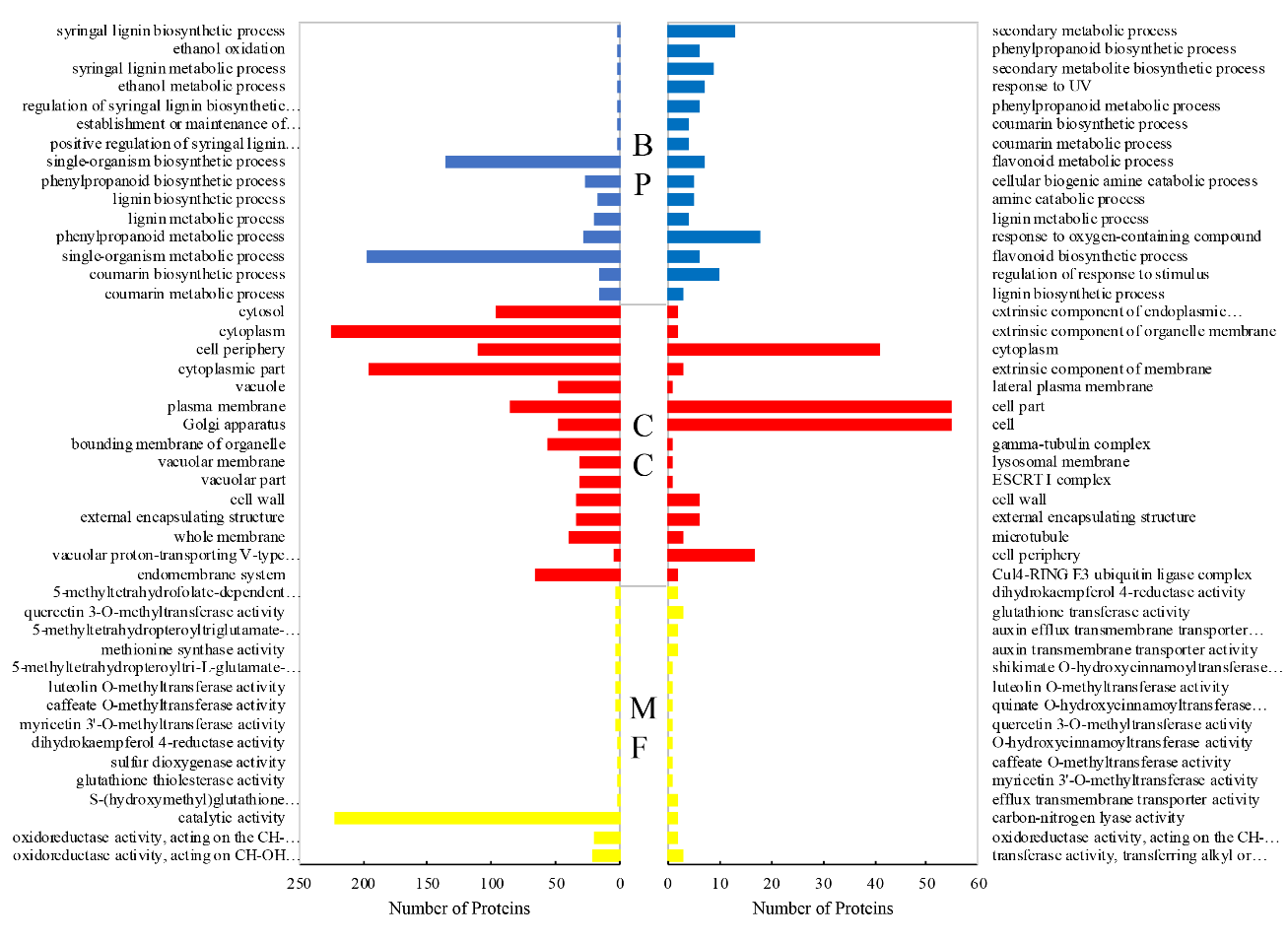


**Fig. S4** GO analysis showing the top10 terms in molecular function, cellular component and biological process.

**Reference**

1. Chen C, Chen H, Zhang Y, Thomas HR, Frank MH, He Y, Xia R: **TBtools: An integrative toolkit developed for interactive analyses of big biological data**. *Mol Plant* 2020, **13**(8):1194-1202.
